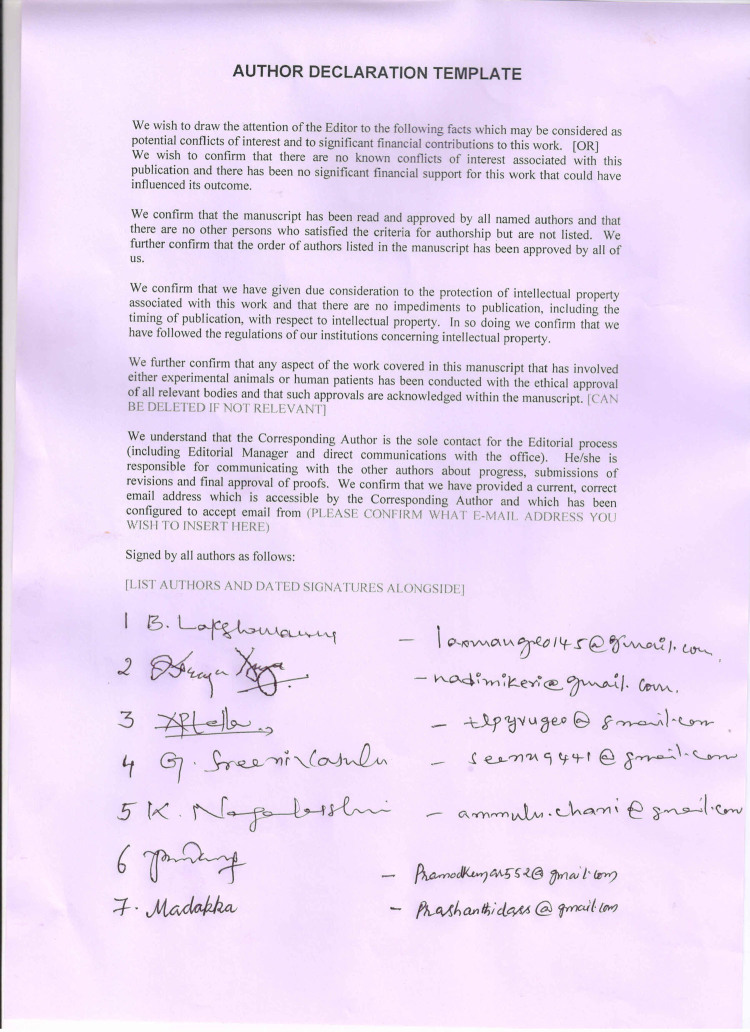# Data on *Molluscan Shells* in parts of Nellore Coast, southeast coast of India

**DOI:** 10.1016/j.dib.2017.11.081

**Published:** 2017-12-05

**Authors:** B. Lakshmanna, N. Jayaraju, T. Lakshmi Prasad, G. Sreenivasulu, K. Nagalakshmi, M. Pramod Kumar, M. Madakka

**Affiliations:** a Department of Geology, Yogi Vemana University, Kadapa 516003, A.P., India; b Department of Earth Sciences, Yogi Vemana University, Kadapa 5160003, A.P., India; c Department of Biotechnology & Bioinformatics, Y.V. University, Kadapa 516003, India

**Keywords:** *Mollusca* shells, Organic matter, FTIR Spectroscopy, XRD, SEM and EDS analysis

## Abstract

X-ray diffraction (XRD), Scanning Electron Microscope-Energy Dispersive Spectroscopy (SEM-EDS), and Fourier Transform Infrared Spectroscopy (FT-IR), were applied to analyze the organic matrix of two *Molluscan shells*. The *Mollusca shells* are mineral structure and calcium carbonate crystallized as aragonite. The FT-IR spectra showed Alkyl Halide, Alkanes, Alcohols, Amides, Aromatic, and Hydroxyl groups in the organic matrix of the whole (organic and mineral) Molluscan shells. SEM images of particles of the two Molluscan shells at different magnifications were taken. The morphologies of the samples show a flake like structures with irregular grains, their sizes are at micrometric scale and the chemical analysis of EDS indicated that the major elements of *Cardita* and *Gastropoda* were C, O, and Ca, consistent with the results of XRD analysis. The results of the analysis of the EDS spectra of the shells showed that the content of most of the powder composition of shells is the element carbon, calcium oxygen, aluminium, and lead peaks that appear on the *Cardita* and *Gastropoda* and shells powders tap EDS spectra. The present work examined organic matrix of the selected shells of the heavily polluted and light polluted sites, along Nellore Coast, South East Coast of India. The heavily polluted sites have significantly thickened shells. The data demonstrated the sensitivity of this abundant and widely distributed intertidal fragile environment.

**Specifications Table**

**Table:** 

**S.No**	**Subject area**	**Palaeontology**
1	More specific subject area	Palaeontology
2	Type of data	XRD, EDS-SEM, FTIR
3	How data was acquired	Experimental
4	Data format	Analysed
5	Experimental factors	*Cardita* and *Gastropod* Shells
6	Experimental features	Heavy pollution, light pollution and Non-polluting
7	Data source location	Nellore Coast
8	Data accessibility	The Data Available with this article

**Value of the data**

• Spectroscopy (X-ray Diffraction (XRD), Fourier transforms Infrared (FTIR)), Scanning Electron Microscope (SEM) and Energy Dispersive Spectroscopy (EDS) were utilized to study the variations in organic groups and elements present in the *Cardita* and *Gastropoda* (*Mollusca shells*).
• Examined malformations among the selected shells of the heavily pollution, light pollution and Non-polluted sites in Nellore coast.
• Data on shell shape, thickness, dry weight, microstructure and semi-quantitative elemental composition was evaluated.

## 1 Data

*Mollusc shells* sometimes include associate degree outer sclerotized macromolecule layer known as periostracum and inner calcified layers. Besides, a hinge system is a gift in *Molluscan* that joins the 2 shells at their dorsal margins. In most prosobranchs, associate degree organic or calcified plate is a gift on the dorsal surface of the met podium of the foot. Polyplacophorans (chitons) disagree from the opposite *Molluscan* categories therein the onerous components consisting of eight shell plates lined by skinny organic material, and spines lined by a cuticle substance [1], [2]. The phylum shell formation emphasizes the physiological method [3]. The Broad-Ribbed *Cardita* could be mistaken for associate degree bivalve (Family *Arcidae*). The adult bivalve is roughly 1½ inches long. The shell is durable, bluffly oval, and has concerning 20 strong diverging ribs. The ribs area unit wide, scaly, and have auburn spots scattered over them in somewhat homocentric bands. The background color of the shell is white, grayish, *Carditas* could have lost their markings. Recent specimens sport a gray periostracum. The within of this sort of clam shell is porcelain-white. The gumbo, of the shell, is giant and is settled a few fourth of the approach from the rounded front of the shell. The correct valve of the claim contains a giant central tooth, and therefore the left valve contains a smaller central tooth. The lunule, the world ahead of the convexity, is depressed and formed sort of a plump Valentine. There's a slim, external ligament that connects the 2 valves. The *Gastropoda* includes most *Molluscs*’, as many as 60,000 existing species and 50,000 fossil forms. The gastropods have associate degree unsymmetrically spiral shell that functions as a conveyable retreat. The body of *Gastropoda* is generally composed of a head, foot, visceral hump, and mantle. Visceral organs show well-organized options including cardiovascular system, an organic process and emission system, and a genital system. Each sexual and hermaphroditic copy area unit found among the varied species and families. The crystallization method of shell by calcium carbonate is considered to be a long progressive activity that depends mainly on several a intrinsic and environmental factors. The fabric of shells is usually being a carbonate. Shell tubules area unit microscopic canals within the shells of varied *Molluscs*. In fissure lid gastropods, the canals type by shell growth around cellular extensions of the mantle epithelial tissue (caeca). Being associate degree edible clam, the *Cardita* and *Gastropoda* area unit sought-after out by many various predators. Predators will embrace octopi and humans. On inside the phylum shells area unit typically a pearly white though some with chromatic tinges and dark grays area unit gift. Phylum shells could are stained by one thing when the animal died or it's been absorbed from the sand wherever the animal was living. It's one thing, however, found them in several colors with the colors "bleeding out" to the within of the phylum shells.

### 1.1 Study area

**Fig. 1:**
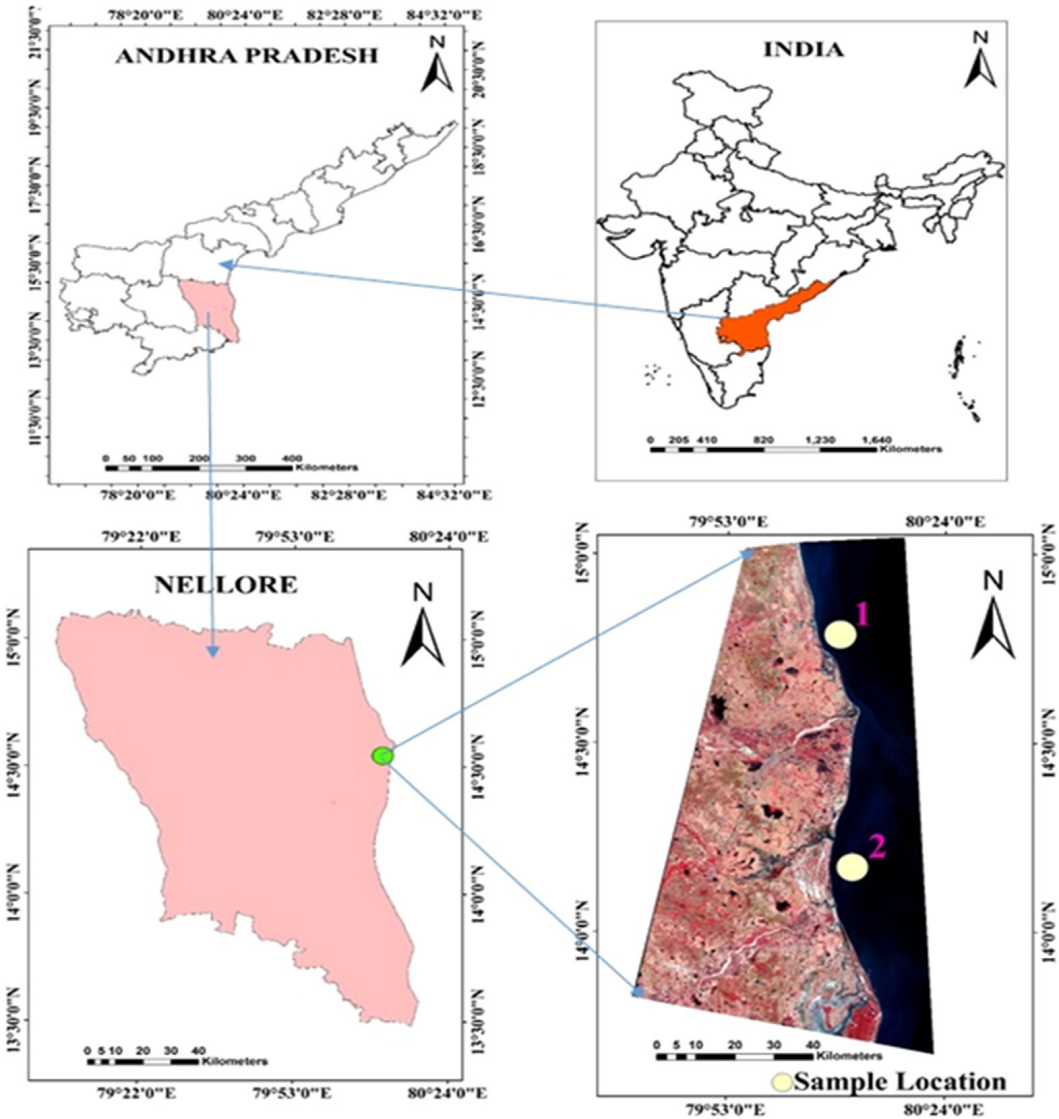
Location of the study area at Nellore coast, Andhra Pradesh, India. (1.*Cardita,* 2.*Gastropoda*).

The study space covers concerning one hundred ten kilometres (Fig. 1). The current investigations area unit thought of to be initial elaborate analysis work ever dispensed on *Mollusca shells* underneath varied pollution outfalls and its implications on the Nellore Coast regarding metals pollution. Such studies area unit solely confined to the *Mollusca shells*. The realm underneath investigation enjoys a tropical sub-humid sort of climate with associate degree annual mean temperature of 29 °C. The summers recorded as high as to 32°, winters 28 °C. The humidness varies from 71% to 73% during summer and 88–90% during winter seasons, and consequently, the speed of evaporation is fairly high. *Gastropoda* and *Cardita* support they're usually accepted shut biological process relationship. In line with J.C. Carter, shell tubules also are a gift in astartids indicating a way wider distribution of this feature within the *Carditoida* than is presently mirrored within the literature. The distinction between epicycloid and sarcoid shell tubules indicates completely different functions and origins, and maybe the involvement of various cell varieties. However, associate degree assessment of this hypothesis needs microscopic anatomy examination of the mantle, that was not out there for the current study.

## 2 Experimental design, materials and methods

**Fig. 2:**
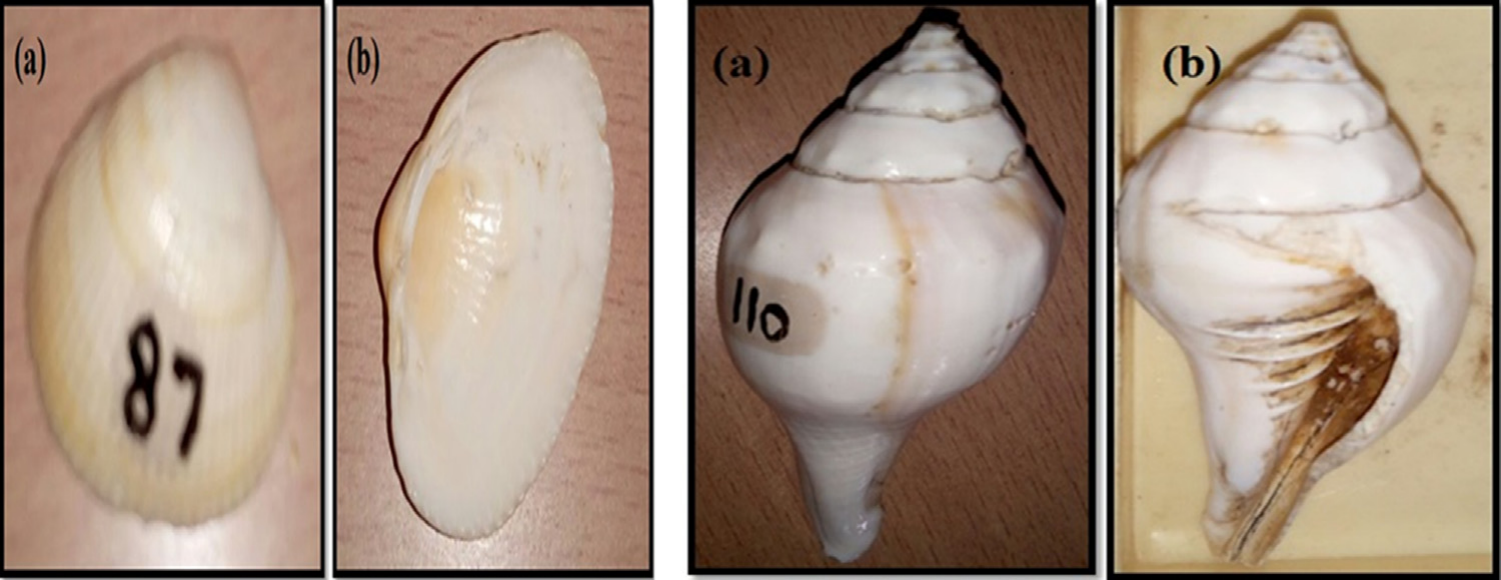
*Cardita* Shell (a) *Dorsal* (b) Ventral, and *Gastropoda* Shell (a) Dorsal (b) Ventral.

The *Mollusca shells* of *Cardita* and *Gastropoda* were collected from some parts of Nellore coast, South east coast of India (Fig. 2). These shells were washed with a pure water, air dried and then pulverized with mortar and pestle for further characterization. The crystal phase analysis was done by Rigaku mini flex 600 X-ray Diffraction (XRD) machine with CuK_α_ (1.5418 Å) wavelength. The surface morphology of the samples and the elemental analysis was investigated by Carl Zeiss EVO ma15 Field Emission Scanning Electron Microscope (FE-SEM) with Energy Dispersive Spectroscopy (EDS) option. Fourier transform infrared (FT-IR) spectra were recorded on a Perkin Elmer Spectrum Two, UK Fourier transform spectrometer.

### 2.1 X-ray diffraction analysis

**Fig. 3:**
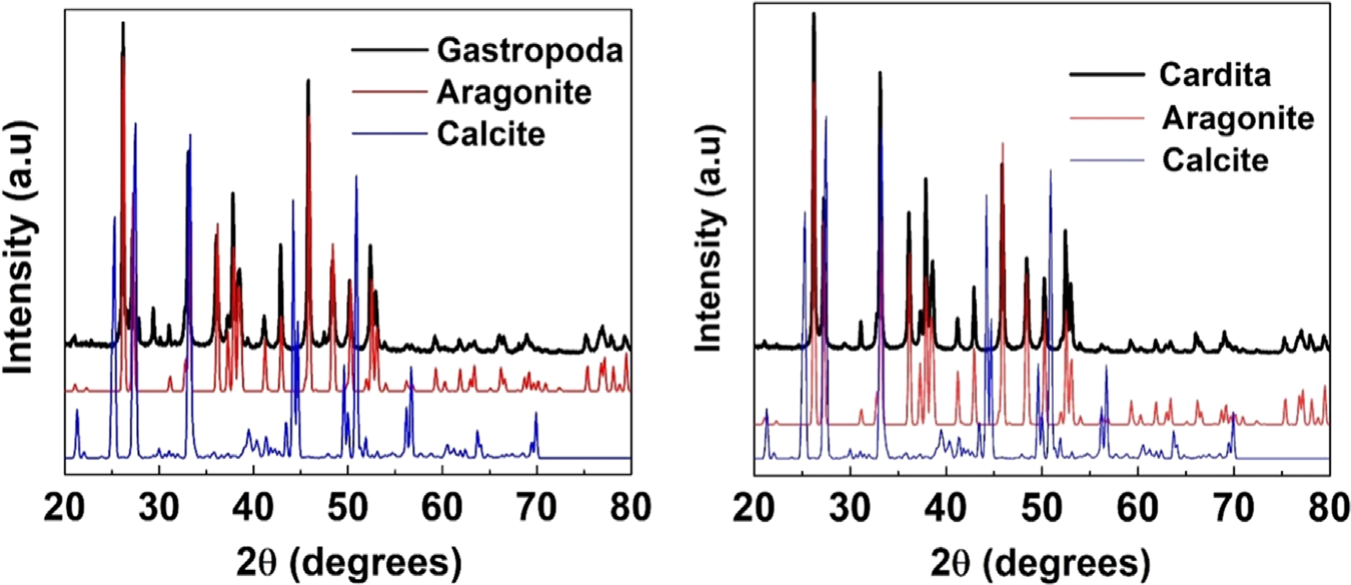
X-ray diffraction analysis of *Gastropoda* and *Cardita*.

The Phase identification of the samples under study are investigated by using the X-ray diffraction (XRD) technique for phase identification. The room temperature XRD patterns of *Gastropoda* and *Cardita* are depicted in Fig. 3 from the figure, it can be seen that the detraction peaks of both the samples were majorly indexed to orthorhombic aragonite phase (CaCO_3_, JCPDS card no. 86-2334) with a minor portion of calcite phase. It is observed that the intensity of the plane at 2θ ~ 29° corresponding to the calcite phase is more for the *Gastropoda* indicating the high percentage of calcite phase in *Gastropoda* compared to the *Cardita*. The lattice parameters and cell volume of the orthorhombic aragonite phase found to be, *a* = 4.9648 Å, *b* = 7.9608 Å, *c* = 5.7497 Å, *V* = 227.25 Å^3^ for *Cardita*, whereas these values increases slightly to *a* = 4.9681 Å, *b* = 7.9690 Å, *c* = 5.7536 Å, *V* = 227.79 Å^3^ for *Gastropoda* respectively. The crystallite size of the samples are calculated using Debye Scherer's formula and the average crystallite size is found to be ~70 nm and ~60 nm for *Gastropoda* and *Cardita.*

## 3 Morphological studies

### 3.1 SEM (*Cardita* and *Gastropoda*)

**Fig. 4:**
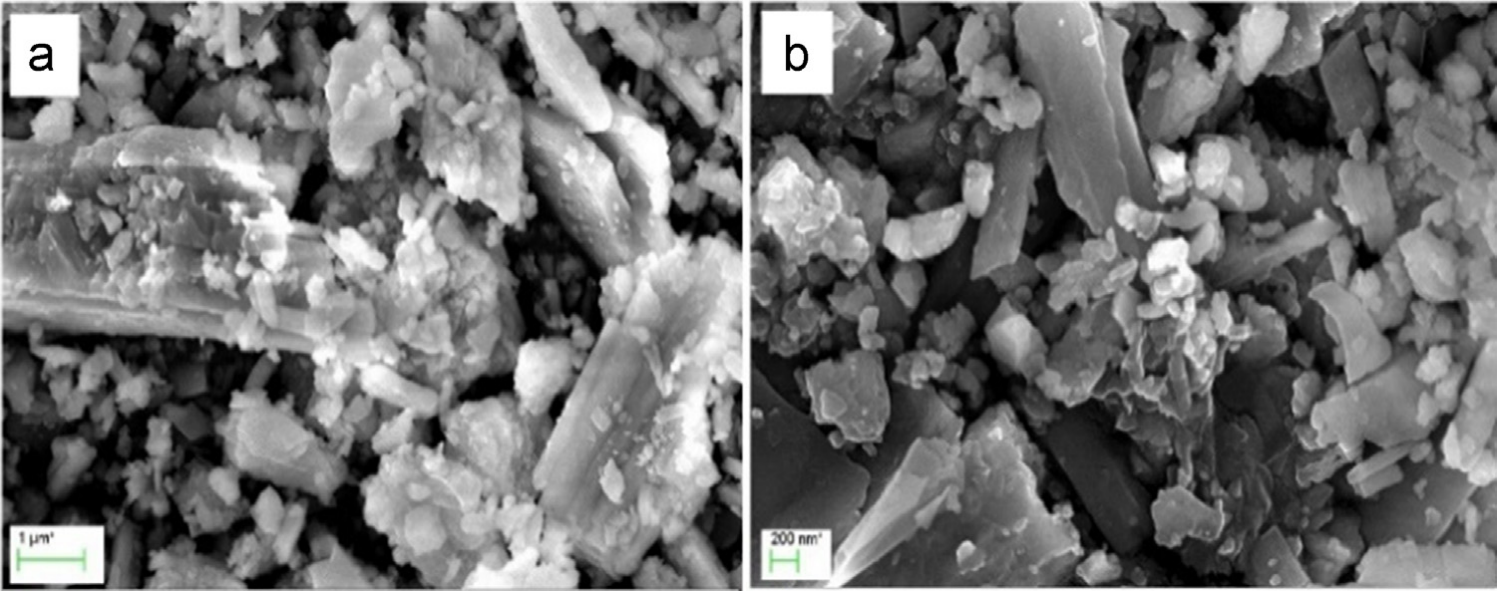
Scanning Electron Microscope images of *Cardita* shell at different magnifications.

**Fig. 5:**
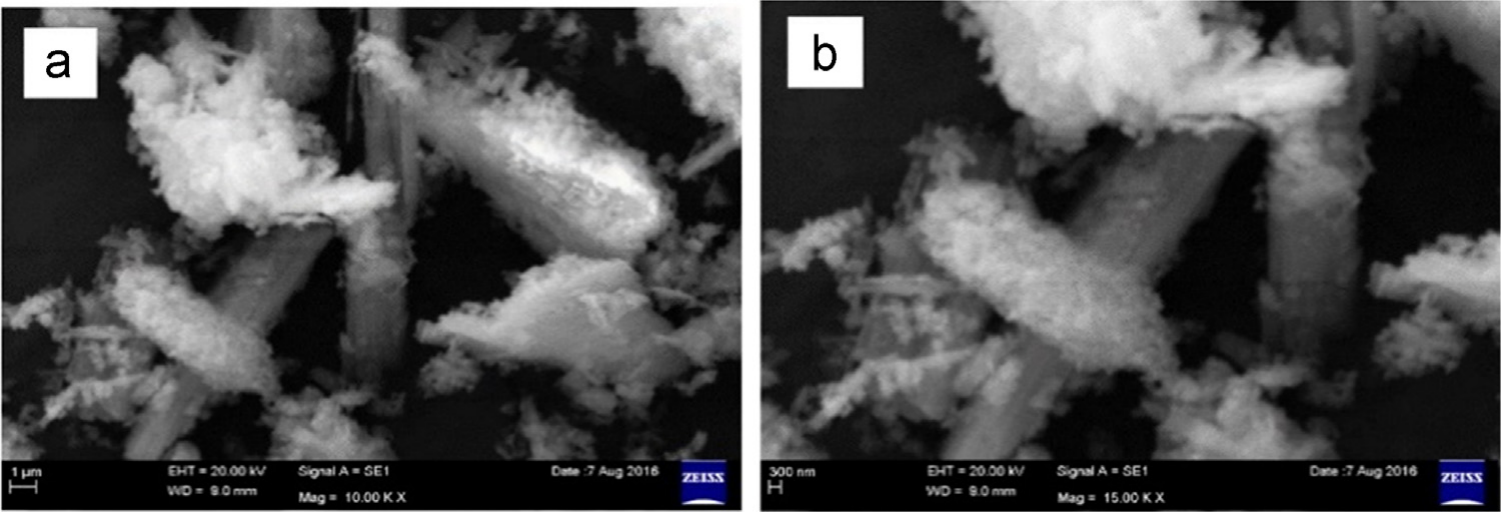
Scanning Electron Microscope images of *Gastropoda* shell at different magnifications.

A Scanning Electron Microscope (SEM) is a facility that produces images of a sample by scanning the surface with a focused beam of electrons. The electrons interact with atoms in the sample, producing various signals that contain information about the sample's surface topography and composition. The electron beam is scanned in a raster scan pattern, and the beam's position is combined with the detected signal to produce an image. The typical SEM images of particles of the two *Molluscan shells* at different magnifications and morphologies of the samples show a flake like structures (Fig. 4, Fig. 5) with irregular grains, whose sizes are at micrometric scale. Furthermore, it is observed that the grain size of the *Gastropoda* is higher compared to the *Cardita*. Moreover, those *Mollusca*n samples showing a well-defined pattern, as evident, the *Molluscan* samples can be divided into two categories: those having a ‘laminar’ pattern, forming mineral phase and this process can occur in two different ways. The first involves mineral precipitation in the open environment, without any apparent control by the cell over the mineral product. This process was defined by as ‘biologically induced bio mineralization,' with mineral forming only as a by-product of the cells metabolic activity or through its interaction with the surrounding aqueous environment. By contrast, the second way, ‘biologically controlled bio mineralization,' is completely regulated, allowing the organisms to precipitate mineral that serves some physiological purpose [1].

### 3.2 Energy Dispersive Spectroscopy (EDS): (*Cardita* and *Gastropoda*)

**Fig. 6:**
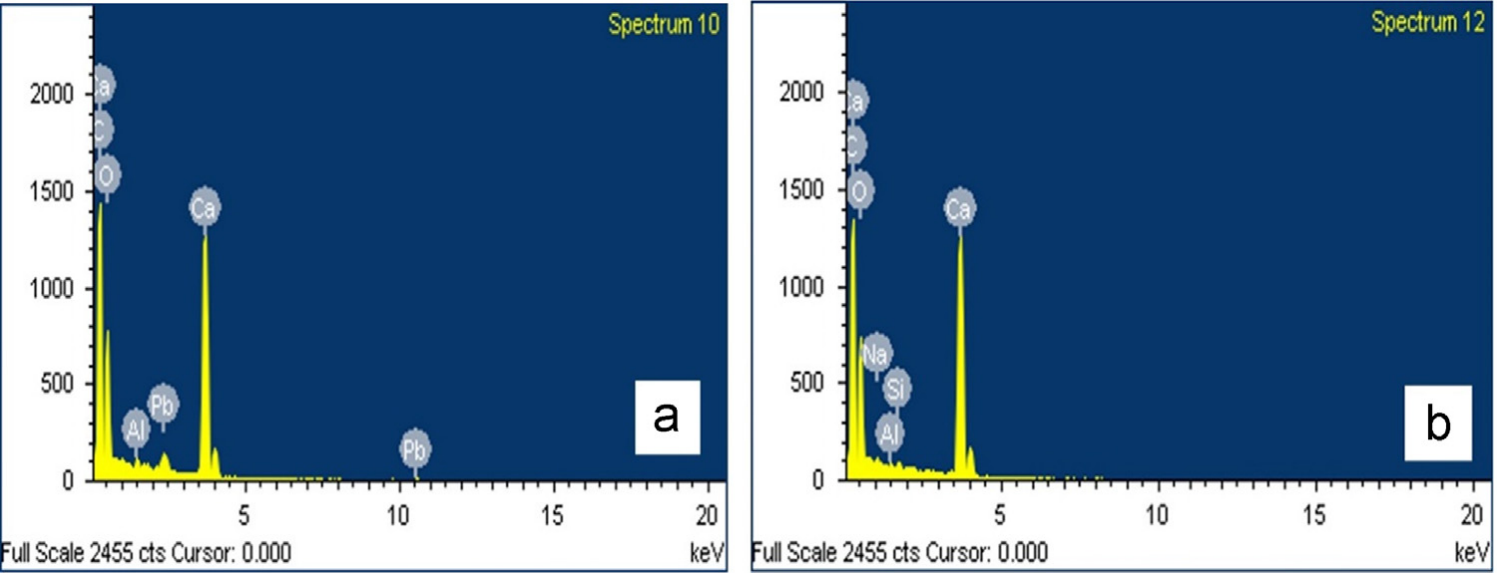
EDS spectra of (a) *Cardita* and (b) *Gastropoda* shells.

The EDS analysis indicated that the major elements (C, O, and Ca) of *Cardita* were, consistent with the results of XRD analysis (Fig. 3, Fig. 6). The results obtained by EDS spectra of the shells shows that the content of most of the powder composition of shells is the element carbon, calcium–oxygen, aluminium, and lead peaks that appear on the *Cardita* shell powders taps EDS spectra that can be seen in Fig. 6(a) and (b). It can be observed that the structure of *Cardita* and *Gastropoda* shells in naturals comprised of carbon (37.64% and 37.87%), oxygen (42.88% and 44.15%), only for this belongs to *Cardita* Aluminium (0.51%), Ca (16.57%), and Pb (2.41%) adsorbed on the surface of the material (Table 1). It is known that the constituent elements of the largest Shell are carbon (C), oxygen and calcium (Ca), which form the compound CaO. The results of EDS analysis shows that the shell raw unbaked *Molluscan shells* (*Cardita* and *Gastropoda*) composed of atomic elements C 50.08% and 49.44%, O 42.83% and 43.27%, Al 0.30%, Ca 6.61%and Pb 0.19%. It can be observed that the structure of *Cardita* shells in natural*s* comprised of carbon (37.87%), oxygen (44.15%), *Gastropoda* only for this belongs to elements Na (0.53%), Al (0.21) Si (0.33) and Ca (16.90%), adsorbed on the surface of the material [2].

**Table 1:** EDS data of *Cardita* and *Gastropoda* shells.

	***Cardita*****(Weight %)**	***Cardita*****(Atomic %)**	***Gastropoda*****(Weight %)**	***Gastropoda*****(Atomic %)**
C K	37.64	50.08	37.87	49.44
O K	42.88	42.83	44.15	43.27
Al K	0.51	0.30	–	–
Ca K	16.57	6.61	–	–
Pb M	2.41	0.19	–	–
Na K	–	–	0.53	0.36
Al K	–	–	0.21	0.12
Si K	–	–	0.33	0.18
Ca K	–	–	16.90	6.61
Total	100	100	100	100

### 3.3 FT-IR spectra analysis

**Fig. 7:**
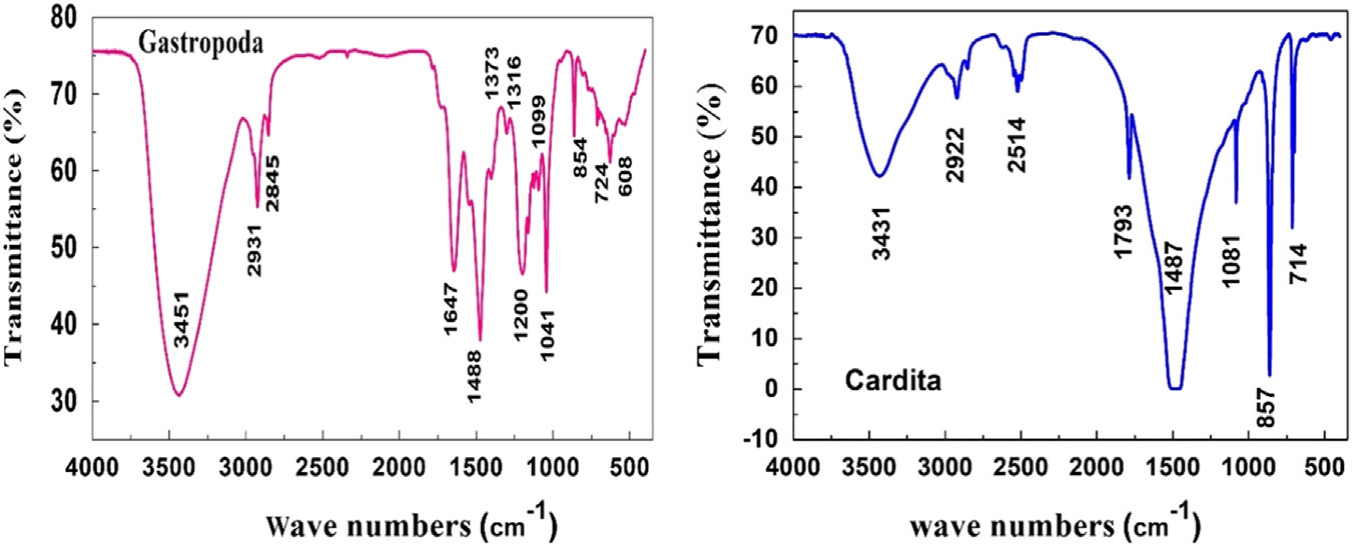
FTIR spectra of *Mollusca shells*: *Cardita* and *Gastropoda*.

Fig. 7 shows the FTIR spectra of *Mollusca shells*: i.e., *Cardita* and *Gastropoda*. In the organic linings of *Molluscan shells*, a band in the region 3451 cm^−1^ and 3431 cm^−1^ arises from the stretching vibration of a hydroxyl group, H-bonded OH stretching was observed at *Gastropoda* and *Cardita*. Alkanes C–H stretching was observed with in the absorption range of 2931–2922 cm^−1^ for *Gastropoda* and *Cardita*. Alkanes C–H stretching was observed with in the absorption range of 2845 and 2922 cm^−1^ for *Gastropoda* and *Cardita*. An absorption band at 1793 cm^−1^ for *Cardita* was observed. Amides; C=O stretching absorption band was observed data peak range 1647 cm^−1^ for *Gastropoda*. The absorption peak at Alkyl Halide C=C stretch 1487 cm^−1^ for *Gastropoda*. The absorption peak at 1316 cm^−1^ Alkyl Halide C–F stretch, the Absorption peak at 1099 cm^−^^1^ and 1081 cm^−1^ corresponds to alcohol C–O stretch. Also, a band in the region 854 cm^−1^ and 857 cm^−1^, arises from the stretching vibration of Alkanes; CH stretch for *Gastropoda* and *Cardita* respectively. The absorption peak at 724 cm^−1^ and 714 cm^−^^1^ belongs to alkyl Halide C–Cl stretch, a band in the region 608 cm^−^^1^ arises from the stretching vibration of an Alkyl Halide, C–Cl stretch in *Gastropoda*. The detailed FTIR frequency wavenumber and its assignment of *Mollusca Shells* are presented in Table 2. [4].

**Table 2:** FTIR frequency wavenumber and its assignment of *Mollusca shells*.

**S.No**	**Frequency wavenumber (cm**^−^^**1**^**)**		**Assignment**
	***Gastropoda***	***Cardita***	
1	608	–	Alkyl Halide C–Cl stretch
2	724	714	Alkyl Halide C–Cl stretch
3	854	857	Alkanes; CH bending
4	1099	1081	Alcohol C–O stretch
5	1316	–	Alkyl Halide C–F stretch
6	–	1487	Alkyl Halide C=C stretch
7	1373	–	Alkanes -C–H Bending
8	1647	–	Amides; C=O stretch
9	–	1793	Aromatic
10	2845	2514	Alkanes; CH stretch
11	2931	2922	Alkanes; CH stretch
12	3451	3431	Hydroxygroup,H-bonded OH stretch

## 4 Conclusions

The organic matrix of *Molluscan shells* is strongly linked to the mineral and has a much greater thermal stability than the organic matrix of bone, as it is vital for the use as a biomaterial for orthopaedic applications. The FT-IR spectra of the whole *Molluscan shell* show that there are several functional groups (Alkyl Halide, Alkanes, Alcohols, Amides, Aromatic, and Hydroxyl group) in the whole material (organic matrix and mineral) and in the insoluble organic matrix. Accumulation of Pb and depositing of Ca as carbonate in the shell take place at constant rates relative to each other. Irrespective of metabolic turn-over, both processes can be regarded as organismic net immobilization of matter which produces increasing amounts with time. It seems attractive to compare pathways and balance of Pb and Ca, the more so since both elements occur as free bivalent ions in the marine environment. Accumulation of metals and integrate the chemical contamination signal over the life of the organism. Refinement of techniques for determining element using bivalves is important if global monitoring is to become a reality. Although the results of the present study since very precise indications on the key differences between the crystal micro morphologies of carbonates of biogenic and abiogenic character.

## Figures and Tables

**Figure:**